# Estimation of Caenorhabditis Elegans Lifespan Stages Using a Dual-Path Network Combining Biomarkers and Physiological Changes

**DOI:** 10.3390/bioengineering9110689

**Published:** 2022-11-14

**Authors:** Yao Song, Jun Liu, Yanhao Yin, Jinshan Tang

**Affiliations:** 1 School of Computer Science and Technology, Wuhan University of Science and Technology, Wuhan 430081, China; 2 Hubei Province Key Laboratory of Intelligent Information Processing and Real-Time Industrial System, Wuhan 430065, China; 3 Department of Health Administration and Policy, College of Public Health, George Mason University, Fairfax, VA 22030, USA

**Keywords:** *C. elegans*, CNN, aging, lifespan stages, microscopic images, imaging

## Abstract

Assessing individual aging has always been an important topic in aging research. Caenorhabditis elegans (*C. elegans*) has a short lifespan and is a popular model organism widely utilized in aging research. Studying the differences in *C. elegans* life stages is of great significance for human health and aging. In order to study the differences in *C. elegans* lifespan stages, the classification of lifespan stages is the first task to be performed. In the past, biomarkers and physiological changes captured with imaging were commonly used to assess aging in isogenic *C. elegans* individuals. However, all of the current research has focused only on physiological changes or biomarkers for the assessment of aging, which affects the accuracy of assessment. In this paper, we combine two types of features for the assessment of lifespan stages to improve assessment accuracy. To fuse the two types of features, an improved high-efficiency network (Att-EfficientNet) is proposed. In the new EfficientNet, attention mechanisms are introduced so that accuracy can be further improved. In addition, in contrast to previous research, which divided the lifespan into three stages, we divide the lifespan into six stages. We compared the classification method with other CNN-based methods as well as other classic machine learning methods. The results indicate that the classification method has a higher accuracy rate (72%) than other CNN-based methods and some machine learning methods.

## 1. Introduction

### 1.1. Biological Background

*Caenorhabditis elegans* is one of the most important invertebrate model organisms in biological research. It has the characteristics of a short life cycle, a simple physiological structure, and a transparent worm body which makes for easy observation. Since the early 1960s, it has been widely used as a popular model organism [1]. Its research spans multiple disciplines, including large-scale gene function and characterization research [2], the complete lineage tracing of whole-body cells, the structural construction of the animal nervous system connection group [3], etc. Adult *C. elegans* are about 1 mm long and live in the soil. Under normal conditions, most are hermaphrodites and a few are males. The proportion of males can be greatly increased under special circumstances. *C. elegans* can grow and reproduce at 12 to 25 °C. In an environment of 25 °C, most *C. elegans* have an average lifespan of 12.1 days, with a standard deviation of 2.3 days of incubation [4]. *C. elegans* also provides an ideal model for studying the variability inducement that leads to differences in individual health and lifespan: the relative variability reflected in the lifespan cycle of about two weeks is almost as great as that of human beings from birth to 80 years old. In recent years, with the application of cutting-edge technologies, such as machine learning and artificial intelligence, in biological research, many researchers have used methods such as deep learning in biological research. Hua et al. developed an end-to-end ECG classification algorithm to help classify ECG signals and reduce the workload of physicians [5]. He et al. developed a novel evolvable adversarial framework for COVID-19 infection segmentation [6]. Mu et al. proposed a progressive global perception and local polishing (PCPLP) network to automatically segment pneumonia infection caused by COVID-19 in computed tomography (CT) images [7]. Zhao et al. developed a deep learning model combining a feature pyramid with a U-Net++ model for the automatic segmentation of coronary arteries in ICA [8]. Liu et al. proposed a new method for nuclei segmentation [9]. The proposed method performs end-to-end segmentation of pathological tissue sections using a deep fully convolutional neural network. Cao and Liu proposed a method for segmenting the terminal bulb of *C. elegans* based on the U-Net network [10]. This method solves the problem encountered with traditional single-stage networks that are not suitable for small samples. Lin et al. presented a quantitative method for measuring physiological age *in C. elegans* using a convolutional neural network (CNN) [11]. Fudickar et al. proposed an image acquisition system [12]. This system was used to create large datasets containing entire dishes of *C. elegans*. At the same time, the authors used the object detection framework Mask R-CNN to localize, classify, and predict the outlines of nematodes.

Regarding the lifespan assessment of *C. elegans*, there are currently two main research directions: one is to use physiological changes for evaluation; the other is to use biomarkers for evaluation. The physiological changes here refer to the physiological changes in *C. elegans* that can be directly observed, such as the swallowing rate of the pharynx, measurement of image entropy, measurement of appearance, measurement of exercise capacity, and measurement of auto-fluorescence. In the study of Zhang et al., based on the view that the aging of *C. elegans* is a plasticity process discovered by predecessors, a large number of comparative experiments were carried out on the aging of *C. elegans* [13]. Experiments have found that there is a big difference in lifespan among individuals of *C. elegans*. Short-lived *C. elegans* have a lifespan of about 12 days, while long-lived *C. elegans* can survive for more than 20 days. Through more detailed experiments, the authors determined the difference between the long-lived and short-lived *C. elegans* in the physiological process of aging. The difference in the lifespan of *C. elegans* is mainly concentrated in the time period between reproductive maturity and death, which is less related to the time of larval growth. Although it takes an average of 2.1 days for larvae to develop (accounting for 17.3% of the average lifespan), the variability in the development time is less than 0.1% of their total lifespan. In the subsequent lifespan process, the physiological health status and the rates of physiological health changes are related to the total lifespan of *C. elegans*. At the same time, it is also to be pointed out that the differences in physiological changes between different *C. elegans* at the same stage are much smaller than the differences in physiological changes between different *C. elegans* at the same absolute time. Stroustrup et al. designed a set of lifespan machines to judge and predict the lifespan of *C. elegans* by detecting the movement status and movement ability of *C. elegans* and achieved better results [14]. Martineau et al. extracted hundreds of morphological, postural, and behavioral features from *C. elegans* activity videos and used support vector machines (SVM) to analyze their direct relationship with *C. elegans* lifespan [15]. Lin et al. presented quantitative methods to measure the physiological age of *C. elegans* with convolution neural networks (CNNs), which measured ages with a granularity of days and achieved a mean absolute error (MAE) of less than 1 day [11]. Furthermore, they proposed two models: one was based on linear regression analysis and the other was based on logistic regression. The linear-regression-based model achieved a test MAE of 0.94 days, while the logistic-regression-based model achieved an accuracy of 84.78 percent with an error tolerance of 1 day. The advantage of using physiological changes for assessment is that this method has a higher accuracy rate and is applicable to various *C. elegans* mutants. However, because the research is limited to the *C. elegans* body and lacks the possibility of technological migration, the significance for human research is relatively limited.

Compared with physiological changes, biomarkers mainly consist of life-related genes or microRNA promoters carrying fluorescent proteins. The relevant signal pathway mechanism behind the genes is clear, and there is the possibility of technology migration, which has potential guiding significance for the assessment of human aging [16]. For example, Wan et al. designed a *C. elegans* life prediction algorithm based on Naive Bayes [17]. They investigated the relationship between *C. elegans* gene sequencing information, protein expression information, and lifespan. They proposed a feature selection method based on Naive Bayes to predict the effects of *C. elegans* genes on biological life. However, obtaining gene sequencing information and protein expression information for *C. elegans* leads to the death of the worms, which is not conducive to the verification of the test set and the further development of research. At the same time, the cost of extracting gene sequencing information from *C. elegans* is expensive, and it is not suitable for repeated experiments. Saberi-bosari et al. also selected biomarkers as the research objects, in order to use the Mask R-CNN [18] algorithm to identify the neurodegenerative sub-cellular processes that appear after the senescence of the *C. elegans* PVD neurons, and they used this information to determine current lifespan stages of *C. elegans* [19]. The biological state was divided into three states: young and old adults, cold-shocked and non-shocked nematodes, and cold-shocked and aged worms. Finally, a classification accuracy of 85% was obtained. However, in actual research, it has been found that the currently used biomarkers have the following two problems. On the one hand, the overall performance of biomarkers is relatively poor, which may be due to the limited influence of a single gene on lifespan [20]. On the other hand, some endogenous genes have a certain evaluative power in the wild type, but they often have poor evaluative potential in specific mutant strains (such as daf-16). This is because the genes used in the evaluation are often limited to specific signaling pathways, and the phenomenon of aging is jointly regulated by multiple signaling pathways [21].

We selected proteostasis as a life-related indicator because most biological activities are dependent on protein function and many life-related signal pathways in *C. elegans* show the regulation of proteostasis. With the aging of *C. elegans*, protein accumulation will gradually increase [22]. At the same time, in the process of human aging, proteostasis is also related to many senile diseases [23], such as Alzheimer’s disease [24], Parkinson’s disease [25], and so on. Intrinsic protein aggregation is a biomarker of aging. Knowing how to regulate it will help understand the underlying mechanisms of aging and protein aggregation diseases [26]. For the detection of proteostasis imbalance, there are also mature visual biomarkers in *C. elegans* metastable protein [27]. In summary, the physiological index of proteostasis change is closely related to aging, involves multiple signaling pathways, is conservative among species, and is easy to detect, so it is a good candidate for lifespan estimation. To minimize the impact on *C. elegans*, the response is closest to the aging process in the natural state. We selected firefly luciferase protein, which has not been reported as being related to pathological processes associated with a variety of metastable proteins. *C. elegans* carrying multiple copies of the firefly luciferase gene will not exhibit premature aging and paralysis phenotypes. This is the first study to use protein aggregation as a biomarker to estimate the lifespan of *C. elegans*.

### 1.2. Convolutional Network Background

In recent years, neural networks, representing an emerging deep learning method, have continuously enabled breakthroughs in various fields. Among them, convolutional neural networks (CNNs) [28], as deep feed-forward neural networks involving convolutional calculations, can better obtain spatial position and shape information from an image. They are widely used in many fields, including object tracking, pose estimation, text detection and recognition, visual saliency detection, action recognition, and scene labeling.

Generally, a convolutional neural network consists of a convolutional layer, a pooling layer, and a fully connected layer. The input image is respectively processed by the convolutional layer, the pooling layer, and the fully connected layer, and the feature map is finally outputted. Limited by computer performance and the acquisition of datasets, the convolutional neural network model was unutilized for decades, until the proposal of AlexNet [29] in 2012, which stimulated the frenzy of convolutional neural network research. In this study, we chose VGG16 [30], Inceptionv3 [31], MobileNetV3 [32], ResNet50 [13], and DenseNet [33] as candidate benchmark CNN models.

Given the shortcomings of the above two methods, we hope to design an evaluation and prediction method to maximize accuracy and have better mobility for future research on the human lifespan. To this end, in this paper, we propose an estimation method for *C. elegans* lifespan stages combining a *C. elegans* biomarker (protein aggregation) and a high-efficiency attention improved network (Att-efficientNet). Compared to using shape and motion trajectory features, this is a new attempt. The dual-path feature fusion model based on deep neural networks can extract local features of *C. elegans* through neural networks and compensate for the loss of global features by calculating fluorescent protein aggregation information and finally output the multi-stage classification results for *C. elegans* lifespan stages. In this paper, we divide the lifespan of *C. elegans* into six stages. This method has basically met the needs of biological research to predict the lifespan stages of *C. elegans*. This work makes several main contributions:

- The study is the first to estimate the lifespan stages of *C. elegans* using the six-stage standard from fluorescence microscope images;
- A dual-path network combining biomarker and physiological changes is proposed to jointly estimate the lifespan stages of *C. elegans* from fluorescence microscope images;
- An Att-EfficientNet was developed to extract physiological changes and a unique fluorescent protein feature extraction technology was developed to calculate protein aggregation degrees;
- We evaluated the proposed method on a dataset with 4593 fluorescence microscope images of *C. elegans* and achieved promising results in lifespan stage estimation compared with several other machine learning methods.

## 2. Methods

### 2.1. Materials

The images used in this article were images of live *C. elegans* carrying exogenous transfection of firefly luciferase fusion protein taken by the Sino-French Joint Laboratory of the School of Life Sciences, Huazhong University of Science and Technology, under a fluorescence microscope. In the previous literature, there is no direct evidence regarding the effect of luciferase overexpression on the lifespan of nematodes. Previous studies regarded the luciferase molecule as a protein homeostasis detection biosensor [34,35,36] and did not discuss whether it causes additional protein homeostasis pressure in the worm. In contrast to pathological infectious proteins that can produce significant characterization changes in nematodes and greatly reduce lifespan and to detection proteins that are restricted to specific tissues, luciferase is independent of the nematode gene regulatory network [37] and has a stable effect on the protein network. The fluctuations in its state have high sensitivity and can be expressed in almost all nematode tissues, so it has corresponding advantages. To further reduce the possible proteostasis pressure on the worm, we improved the multi-copy strain used in previous studies using single-copy cloning technology for lifespan prediction. We must admit that due to the metastability of the luciferase protein molecule, nematode individuals may experience prolonged lifespans due to microprotein stress due to its stimulation [34] or shortened lifespans due to the deterioration of protein homeostasis [37]. Therefore, compared with wild-type lifespan experiments, even if there is no significant difference in lifespan, it may be due to the superposition of the two, and potential differences in the regulation of protein homeostasis cannot be ruled out. Therefore, we accepted these limitations and focused on the correlation between protein homeostasis and lifespan itself.

**Figure 1:**
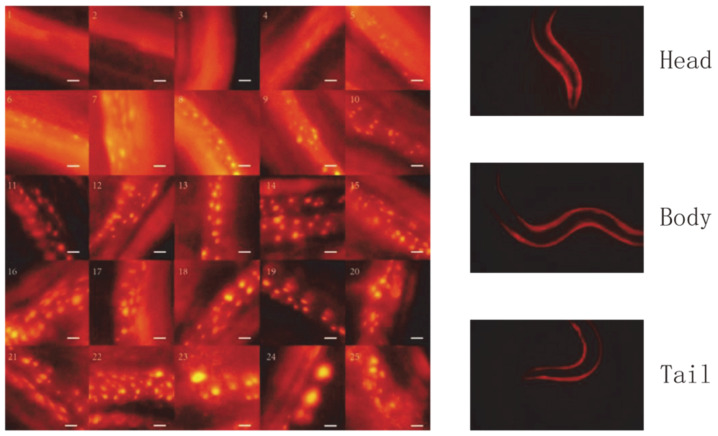
Representative pictures from each class. The serial number in the upper-left area of each picture is the class each picture belongs to. Each class represents a 4% period of time of the whole lifespan of *C. elegans* (e.g., the picture taken on day 5 of an individual with a 15-day lifespan would be assigned to class 9). The original photo was 4000 × 6000 pixels. We manually intercepted 300 × 300 pixels of the region with protein aggregation. The pictures in the figure are representative for each class (not from the same individual). Scale bar: 5 μm (the actual length of the white line is 5 μm).

The images used were from 26 batches of about 40 *C. elegans* each. Images were taken of the head, tail, and torso of *C. elegans* and were evenly distributed across each life stage of *C. elegans*. Each *C. elegans* was cultured independently in a single Petri dish at the L4 stage and photographed daily from the first day after adulthood. A total of 4593 image samples were obtained, and 80% of the images were used as the training set and 20% of the images were used as the test set. In order to obtain accurate lifespan data for *C. elegans* and to try to avoid inaccurate lifespan data for the *C. elegans* caused by damage to the worms during the shooting process, the *C. elegans* were alive at the time the images were taken and no immobilization measures were taken. At the same time, in order to obtain a clear fluorescent protein bright spot during shooting, exposure times of 1/20 s, 1/40 s, and 1/80 s were used. Most previous research on lifespan perception tended to focus on short-term worm phenotype observation. Most of the worms were cultured at 25 °C. Under this condition, *C. elegans* lifespan was mostly concentrated within 15 days. Using the remaining days as an indicator can allow better distinguishment of the degree of aging. However, when cultured at 20 °C, there is a huge difference in the lifespans of *C. elegans* of the same genotype, ranging from 10 to 30 days, with a wider lifespan distribution and lower concentration. If this indicator is used, there will be too great a difference in the actual degree of aging within the same group. For example, a *C. elegans* with a lifespan of 25 days has a remaining lifespan of 5 days at 20 days. *C. elegans* with a lifespan of only 10 days are still in the egg-laying period on the 5th day and have only 5 days of remaining life. Obviously, there is a huge difference in physiology. The other method is to classify directly by the number of days of age, but this method can only assess the age of *C. elegans* and cannot assess the actual degree of aging. To solve such problems, we determined the lifespan of each individual worm in the image set and divided each of the lifespans into 25 stages, 0–4% being the first group, 4%–8% being the second group, and so on. For the *C. elegans* in the dataset, we achieved knowledge of their ultimate lifespans. The assumption behind this method of dividing lifespans according to the proportion of life processes undergone is that the steady-state modes of aggregation and formation of various *C. elegans* proteins are similar, only the rates are different. This assumption can facilitate data division and processing. When the data were insufficient, we often combined different groups. At this time, dividing by proportion can be shown to be convenient, as is shown in Figure 1. In this paper, we divided the dataset into 6 groups, which were merged from the 25 stages. These groups included early, middle, and late stages, and each period included two groups. The 6 groups were formed by combining groups 1–5, 6–9, 10–13, 14–17, 18–21, and 22–25. The number of data in each category in the divided dataset is shown in Table 1. The dataset is shown in Figure 1.

### 2.2. Model Architecture

#### 2.2.1. CNN Model

The effective feature areas in the *C. elegans* images are small, and the images of *C. elegans* at different life stages have close similarities. Compared with natural image classification tasks, *C. elegans* image classification tasks involve more attention being paid to feature information at a fine-grained level. In a traditional convolutional neural network, when the characteristic information is transmitted between the convolutional layer and the fully connected layer, there is some loss of information. Increasing the network depth [38] is a frequently used method for training many neural networks because it can allow the capture of richer and more complex features and adaptation to new tasks for learning. However, increasing the depth of the network will bring about the problem of gradient disappearance. The width of the network is the number of channels in the feature map. Increasing the width of the network [39] means that the number of channels in the feature map increases, and more convolution kernels can obtain more rich features, which enhances the characterization ability of the network. Small-size models require less network width, and wider networks can often learn richer features and are easier to train. However, it is difficult for networks with too wide network structures and shallow depths to learn higher-level features in the process of feature extraction. Convolutional neural networks can also capture fine-grained features for high-resolution input images, which can enrich the receptive field of the network to improve the network.

EfficientNets are a series of models (EfficientNet-B0 to B7) that are obtained by scaling up the basic network (usually called EfficientNet-B0). For all dimensions of the network—width, depth, and resolution—a composite scaling method is used. EfficientNets have attracted much attention due to their advantages in performance. This series of models have surpassed all previous convolutional neural network models in terms of efficiency and accuracy. The width refers to the number of channels in any layer, the depth refers to the number of layers in the CNN, and the resolution is related to the size of the image. To systematically expand the size of a network, composite scaling uses a composite coefficient, which controls how many resources are available for model scaling, and the dimensions are scaled in the following way through the composite coefficient:

(1) $$\begin{array}{l} \mathrm{Depth}:\;d=\alpha^{\phi }\\ \mathrm{Width}:\;w=\beta^{\phi }\\ \mathrm{Resolution}:\;r=\gamma^{\phi }\\ s.t.\alpha \cdot \beta^{2}\cdot \gamma^{2}\approx 2\\ \alpha \geq 1,\beta \geq 1,\gamma \geq 1 \end{array}$$

EfficientNet successfully scales the classification model in three dimensions through the scaling factor and adaptively optimizes the network structure. In this way, during the training process, the training parameters are greatly reduced, and the computational complexity is also reduced.

In this paper, EfficientNet was used for feature extraction from the images of *C. elegans*, and the network is expressed as follows:

(2) $$N=\underset{i=1,2,\cdots ,s}{⊗}F^{L_{i}}\left(X_{\left[H_{i},W_{i},C_{i}\right]}\right)$$

where *N* represents classification network, ⊗ represents the convolution operation, X represents the input tensor, F represents the basic network layer, i represents the number of convolution layers, and $L_{i}$ represents the depth of the network. The network adjusts 3 dimensions (height (H), width (W), and number of channels (C)) for optimization. It is necessary to find the optimal scaling parameters in 3 dimensions. When the model parameters and the amount of calculation are maximized, the accuracy of the model is improved. The maximum accuracy of the model is denoted as ${\mathrm{Acc}}_{\max}\left(N\left(d,w,r\right)\right)$; the specific formula is as follows:

(3) $$\begin{array}{ll} N\left(d,w,r\right)= & ⊗{\hat{F}}^{d\times {\hat{L}}_{i}}\left(X_{\left[r\times {\hat{H}}_{i},r\times {\hat{W}}_{i},w\times {\hat{C}}_{i}\right]}\right)\\ & i=1,2,\cdots ,s \end{array}$$

where depth $d=\alpha^{\varphi }$, width $w=\beta^{\varphi }$, and resolution $r=\gamma^{\varphi }$. The relationship between the variables α, β, and γ is:

(4) $$\alpha^{2}\times \beta^{2}\times \gamma^{2}\approx 2,\alpha \geq 1,\beta \geq 1,\gamma \geq 1$$

**Figure 2:**
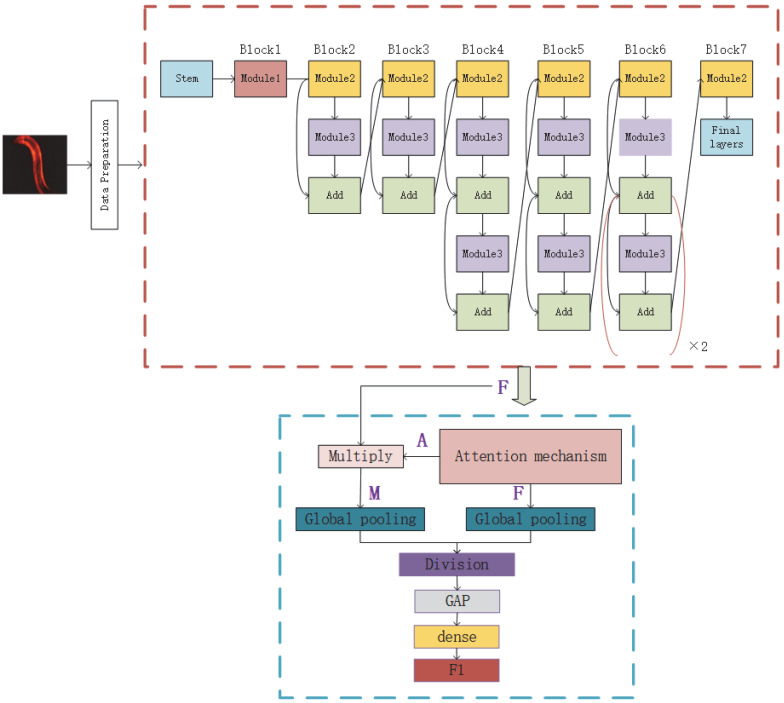
The EfficientNetB0 network is outlined by red dashed lines. Similar to the original EfficientNet model, Stem is a common structure. Module1 contains DepthwiseConv2D, BatchNormalization, and an activation function. Module2 consists of two module1 units which are connected by the Zero Padding layer in the middle. Module3 consists of a global pooling layer, rescaling, and two Conv2Ds. The attention module is outlined by blue dashed lines.

To obtain the three-dimensional parameters which can satisfy Formula (3), the composite parameter φ was used to optimize the depth, width, and resolution of the network. First, we set φ=1, then we found the optimal α, β, and γ parameters satisfying Formula (4) through a grid search. After the experimental adjustments, we obtained α=2.3, β=1.5,and γ=1.18. With Formulas (2)–(4), EfficientNet was used to extract image features, and the features of *C. elegans* images were fused in multiple dimensions. The *M*1 sub-model designed to achieve the above processing is shown in Figure 2.

#### 2.2.2. Attention Mechanism

**Figure 3:**
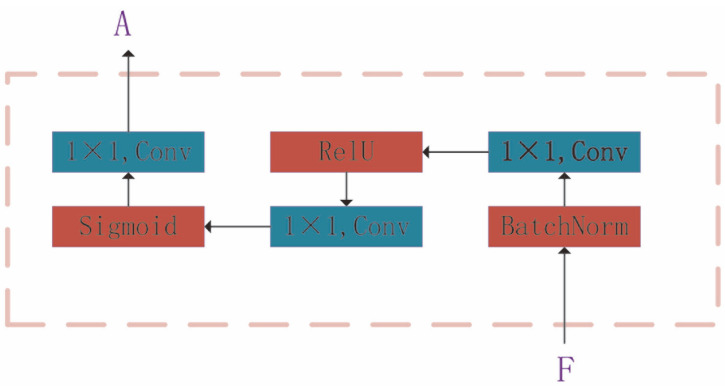
Detailed structure of the attention module. The attention mechanism network mainly takes the output feature map F of the efficient net as input, which is then put into three 1 × 1 convolutional layers. The activation functions ReLU and Sigmoid are added after the batchnorm operation to convert the input into nonlinear features.

As the images of *C. elegans* contained a lot of noise, some images presented problems, such as ghosting, which may interfere with decision making. For example, in live-cell imaging, to prevent damage to cells caused by light, low-light and long-exposure shooting methods are usually used, which make the signal-to-noise ratios of fluorescent bright spot microscopic images low, and thus fluorescent bright spots are difficult to detect, even for experienced biologists. To solve this issue, an attention mechanism was introduced into the network. With the attention mechanism added, the network is able to automatically select the area that needs attention when performing feature extraction. The attention mechanism network mainly takes the output feature map F of EfficientNet as the input and then puts it into three 1 × 1 convolutional layers and adds the activation functions ReLU and Sigmoid to convert the input into nonlinear features. The details are shown in Figure 3.

The purpose of joining this network is to generate an attention map A. Multiplying the feature map F and the attention map A will generate a mask M of the image. To reduce the parameters of the network and avoid overfitting, global average pooling (GAP) is used on the image mask *M* and the attention map A. Finally, the division operation is used to obtain the weight of the image and to filter out irrelevant information. The output of the attention mechanism is:

(5) $$O=GAP\left(A^{l}\right)/GAP\left(A^{l}\times F^{l}\right)$$

where $A^{l}$ and $F^{l}$ represent the l-th layer attention map and the l-th layer feature map, respectively. Before entering the *M*1 module, a *C. elegans* image needs to be averagely pooled to compress the resolution of the image from 6000 × 4000 to 600 × 400 pixels.

### 2.3. Fluorescent Protein Feature Extraction

The feature vector obtained from the *C. elegans* image through the *M*1 module contains rich semantic information, while the macro-level information, such as ROI contour information, is relatively rough. A lot of smaller fluorescent protein spots will be lost because the image needs to be averagely pooled before entering the *M*1 module (the resolution of the image will be compressed from the original size of 6000 × 4000 pixels to 600 × 400 pixels). For feature extraction from a *C. elegans* image, although the abstract high-level semantic information is important, features such as the protein distribution density of the *C. elegans* cannot be ignored either. Therefore, traditional image feature extraction algorithms are employed to extract features based on the density of the spots in the image, which will be fused with the features from the *M*1 module to construct the final feature vector for lifespan prediction. In the microscopic images of *C. elegans*, because the bright spots of fluorescent protein have strong correlations with the lifespan stages of the *C. elegans*, we first detect the spots and then extract the features from the detected spots.

We developed an image processing technique to detect the spots. Since long exposure during image acquisition could cause noise and blur the image, the acquired images are preprocessed before the spots are detected. The preprocess includes two steps: histogram equalization and noise reduction. Histogram equalization is employed to enhance the image and a low-pass filter is employed to reduce the noise. After preprocessing, spot detection is performed. From experiments, we found that the bright spots of the fluorescent protein belong to the high-frequency parts of images, while *C. elegans* tissues and the surrounding backgrounds belong to low-frequency regions. Thus, a high-pass filter is employed to remove the tissue regions and surrounding backgrounds from the images. To high-pass-filter an image, the image is transformed to the frequency domain and then inverse transformed back to the spatial domain after being passed through a high-pass filter. The filtered image is used to detect the fluorescent protein spots. A watershed spot detection algorithm based on local extrema is used.

**Figure 4:**
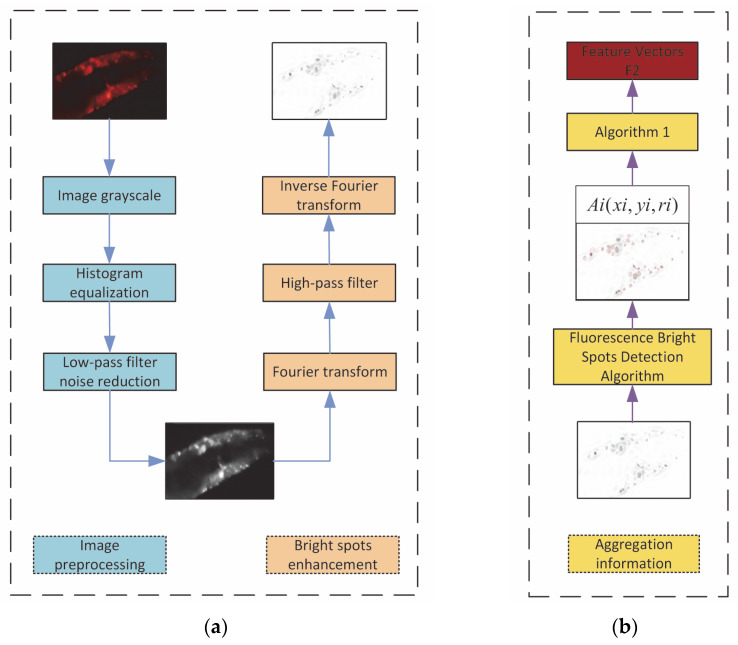
Informational route for fluorescent protein bright spot aggregation degree. (**a**) Image preprocessing. (**b**) Bright spot information extraction and aggregation degree calculation.

OpenCV [40] provides a function called SimpleBlobDetector, which is used to achieve the task. SimpleBlobDetector can screen and mark irregular spots of a specified size and with a limited grayscale range. It has a high detection accuracy through the setting and adjustment of its parameters. As the shape size and grayscale of the fluorescent protein spots in a *C. elegans* image are relatively uniform and the background noise in the image is almost eliminated after low-pass filtering, the algorithm can achieve good results. For the experimental data, the settings for the parameters of the SimpleBlobDetector function are shown in Table 2. The settings were obtained from our experimental tests. The parameter ThresholdStep represents the step value which is the span of the threshold when the algorithm starts to perform threshold segmentation and was set to 8. minThreshold and maxThreshold are the parameters that control binarization, and they were set to 0 and 255, respectively. The parameters minArea and maxArea were set to 10 and 2500; they are used to describe the areas of the spots. minCircularity represents the minimum roundness of a spot, and its value is obtained using the formula $4\pi \left({\mathrm{area}/\mathrm{permeter}}^{2}\right)$. When the roundness is 1, this means that the shape of the spot is a perfect circle; when the roundness is 0, this means that the shape of the spot is a gradually elongated polygon. The minConvexity parameter is the value describing the minimum convexity of a spot, and the formula is area/area of ConvexHull. minInertiaRatio describes the minimum inertia rate of the spot, which is the ratio of the minimum diameter to the maximum diameter of the ellipse. If maxInertiaRatio is not specified, the default value is 1. These parameters describe the basic characteristics of the *C. elegans* fluorescent protein bright spots and can be used to determine whether a blob is a spot or not. SimpleBlobDetector will return the coordinates and radius of each spot, and we denote the i-th protein spot by Ai(xi,yi,ri), where i,=1…,N and N is the number of spots detected. (xi,yi) are the coordinates of the i-th protein spot and ri is the radius of the spot. After we have obtained the coordinates and radius of each spot, we can use them to compute the distance between any two spots. For N spots, we will obtain (N(N−1))/2 distance values $C_{j}$. The distance values $C_{j}$ will be used to construct an n-dimensional feature vector F2=(P1,P2,P3,…,Pn). The algorithm for the computation of Pi and the construction of F2 is described in Algorithm 1. In the experiments, we only computed the value of Pi when i was less than 12. If *i* was greater than 12, Pi took the maximum value of 5000. The algorithm used to extract the features from the aggregation of the fluorescent protein bright spots is shown in Figure 4.
**Algorithm 1.** Calculate Aggregation information *pi***Input: *y* = *Ai*(*xi*, *yi*, *ri*)∨*n*//*Ai*(*xi*, *yi*, *ri*):Fluorescent protein bright spot coordinate set, n: the number of clustering features.****Output: feature vector containing aggregation degree information *F*2**Ensure: *F*2//Feature vector containing aggregation information1:*F*2 <= *Initialize*(*F*2)//Randomly initialize *F*22:**for***i* = *0*, *1*, …, *n* do//n iterations3:  *p* <= Traverse(*Ai*(*xi*, *yi*, *ri*))//Traverse the fluorescent protein bright spot coordinate set (*Ai*(*xi*, *yi*, *ri*))4:**if***p* > 2 then5:  =>9//Go to step 96:**else**7:  =>118:**end if**9:  C*_j_* <= *Dist*(*Random*(*Ai*(*xi*, *yi*, *ri*))//Calculate the distance between any two points in set *Ai*(*xi*, *yi*, *ri*), getting point spacing set C*_j_*10:**end for**11:C*_j_* = Sort(C*_j_*))//Sort the point spacing set C*_j_* from smallest to largest12:M Number(C*_j_*)//The amount of data in the point spacing set C*_j_*13:**if***M* > *n* then14:  =>1815:**else**16:  =>1917:**end if**18:*F*2 (*F*2, …, C*_j_*)//Add all items of point spacing set C*_j_* to the end of feature vector *F*219:*F*2 (*F*2, …, 5000)//Add the set maximum value of 5000 to the end of the feature vector *F*2

**Figure 5:**
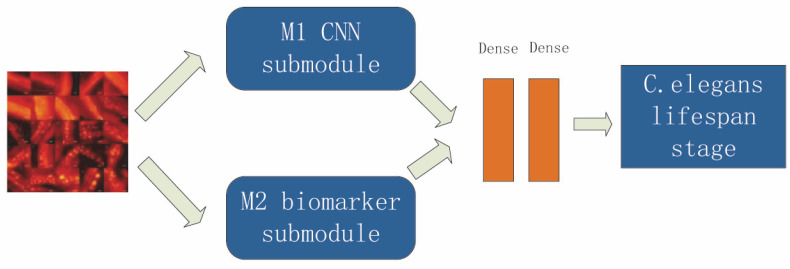
Overall framework for the CNN dual-path feature fusion model.

In this paper, a convolutional-neural-network-based two-way feature fusion model is proposed to solve the problem of the estimation of *C. elegans* lifespan stages. A new additional attribute—aggregation information—is introduced as a global feature to improve classification accuracy. The overall framework of the model is shown in Figure 5, which is divided into two main modules: the CNN feature extraction module M1 and the aggregation feature extraction module M2. The sub-module M1 is an EfficientNet network with an attention mechanism. The feature vector F1 is obtained after the last layer through global average pooling. After the M2 module, the feature vector F2 is obtained. The feature vector F1 is concatenated with the feature vector F2, and finally the evaluation results for the *C. elegans* lifespan stages are outputted through the Softmax classifier [41].

### 2.4. Model Setups

To prevent network overfitting and improve the prediction performance of the test set, dropout was added to the dense layers [42]. This method can randomly eliminate the units in a network during the training process, reduce the errors in the network results introduced by local features, and make the model more robust. Early stopping and fine-tuning were applied to the model to shorten the training and debugging time and improve the performance of the model. In addition, when training deep learning models, the choice of the optimizer is also very important. A good optimizer can significantly speed up the training process while avoiding partial optimization of the results. In this paper, the Adam optimizer with a good adaptive learning rate was used to optimize the classification model.

Regarding Table 1, where $y^{\left(i\right)}\in \left\{1,2,\ldots ,k\right\}$, k is determined according to the number of categories in the actual training dataset, which was taken as 6 in this paper. For a given test input x, the output is a k-dimensional vector representing the probabilities that a sample belongs to each category. The formula is:

(6) $$h_{\omega }\left(x\right)=\left[\begin{array}{c} p\left(y^{\left(i\right)}=1|x^{\left(i\right)};\omega \right)\\ p\left(y^{\left(i\right)}=2|x^{\left(i\right)};\omega \right)\\ \vdots \\ p\left(y^{\left(i\right)}=k|x^{\left(i\right)};\omega \right) \end{array}\right]=\frac{1}{\sum_{j=1}^{k}e^{\omega_{j}^{T}\left(i\right)}}\left[\begin{array}{c} e^{\omega^{T}x^{\left(i\right)}}\\ e^{\omega^{T}x^{\left(i\right)}}\\ \vdots \\ e^{\omega_{k}^{T}x^{\left(i\right)}} \end{array}\right]$$

where $\omega_{1},\omega_{2},\ldots ,\omega_{k}\in {ℛ}^{n+1}$ are the weighting parameters. The coefficient product term on the right side of the formula normalizes the probability distribution so that the sum of the probabilities for all categories is 1.

When using a convolutional neural network to train a model, choosing an appropriate loss function can improve the accuracy and robustness of the model. The two-way feature fusion model proposed in this paper is a multi-class network model, so Categorical Cross Entropy [43] was used as the loss function. The true label of the i-th sample is $y_{ji}$; the predicted value label is ${\hat{y}}_{ji}$.

The hardware configuration used in the experimental environment of this paper was an i7-8700K processor, the GPU model was NVIDIA GeForce GTX 1080, and the video memory was 8 GB. The operating system was Ubuntu Server 16.04 64-bit, and the programming language used was python 3.6.0.

## 3. Experiments and Results

### 3.1. Evaluation Metrics

In the experiments, we used Accuracy (*AC*), Recall, *F*1 score, and a confusion matrix to evaluate the results; the formulae are as follows:

(7) $$AC=\frac{TP+TN}{TP+TN+FP+FN}$$

(8) $$Recall=\frac{TP}{TP+FN}$$

(9) $$P=\frac{TP}{TP+FP}$$

(10) $$F1=\frac{2PR}{P+R}$$

where *TP* means true positive, which means that the prediction is positive and the actual is also positive; *FP* means false positive, which means that the prediction is positive but the actual is negative; *FN* means false negative, which means the predictive is negative but the actual is positive; and *TN* refers to samples that are predicted to be negative and actually negative. The confusion matrix is a kind of evaluation classification model used to subdivide categories.

### 3.2. Experiments

#### 3.2.1. The Influence of *M*1 and *M*2

**Figure 6:**
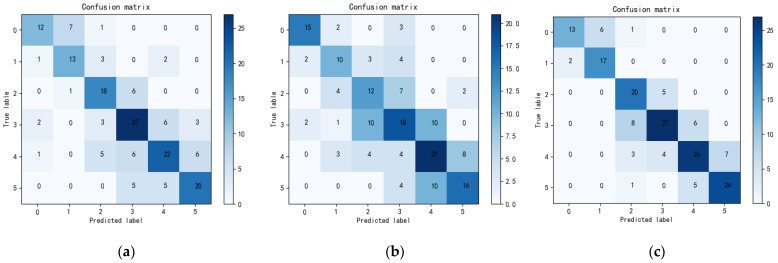
Confusion matrix. (**a**) *M*1 module classification effect. (**b**) *M*2 module classification effect. (**c**) The classification effect of the method in this paper.

To analyze the influence of different network branches in the model, we compared the performance of the networks with or without the M1 sub-module or the M2 sub-module. Three networks were created: the network with both the M1 sub-module and the M2 sub-module (denoted by M1+M2), the network with only the M1 sub-module, and the network with only the M2 sub-module. The experimental results are shown in Table 3. It can be seen that each sub-module has a certain independent classification ability. Figure 6 shows the confusion matrix using the network with only one sub-module and the complete model (M1+M2) of this paper. Compared with the network with only the M2 sub-module, the network with only the M1 module showed a better performance in classification. However, the M1+M2 dual-path method achieved an accuracy score of 0.726, a recall of 0.724, and an *F*1 score of 0.740, which scores are higher than those of both of the networks with only one module. These results show the effectiveness of the proposed dual-path method.

#### 3.2.2. Benefits of the *M*2 Module

We further investigated the performance of lifespan stage estimation with different popular networks by adding the M2 module. The investigated networks were VGG16, InceptionV3, MobileNetV3, ResNet50, and DenseNet. During the experiments, we first replaced the M1 module in Figure 5 with a network listed above and obtained a new hybrid model. After that, the obtained hybrid model was trained, and the trained model was used for the estimation of lifespan stages. For each network, we did the same. The results are shown in Table 4. In Table 4, it can be seen that the hybrid model can generate more accurate results than networks without the M2 module. These results suggested the effectiveness of the proposed protein aggregation degree strategy used in the M2 sub-module. Moreover, it can be seen that all the hybrid models can obtain higher accuracy than the original networks, which indicates that the M2 module proposed in this paper has a certain degree of versatility.

#### 3.2.3. Influence of the Attention Mechanism

To further verify the effectiveness of the attention mechanism with respect to classification networks, the attention mechanism was also added to the VGG16, InceptionV3, MobileNetV3, ResNet50, and DenseNet networks, and the resulting classification result data are shown in Table 5. It can be seen from Table 5 that after the attention mechanism was added more attention could be paid to the main features in the image during classification, which played a positive auxiliary role in the feature extraction of the network. When the attention mechanism was added to EfficientNet, the accuracy rate was increased by 3.5%, which was higher than the result of 0.691 obtained without the attention mechanism, and the classification performance was improved. For other networks, after the attention mechanism was added the classification effect was also improved. However, the improvement of the proposed EfficientNet with the attention mechanism was slightly higher than the improvements achieved with the other classic neural network models.

#### 3.2.4. Comparison with Random Forests and Support Vector Machines

Our method combines traditional machine learning methods and deep learning methods. In traditional machine learning models, random forests and support vector machines are common machine learning methods, and we compared them with the proposed network. It should be pointed out that, in the comparison, the information inputted to the two models only contained the numerical information after the blob processing, not the picture information. The performances are shown in Table 6 and Table 7. From the tables, it can be seen that the performance of the random forest model was generally slightly lower than that of the proposed model, and it performed poorly especially in the 3–5 lifespan stages. The results for the support vector machine model were generally not ideal, and the classification accuracy in stages 5–6 was 0. Overall, the proposed network showed significant advantages over the traditional machine learning methods.

## 4. Discussion and Conclusions

In this study, we explored deep learning methods for the assessment of lifespan stages of *C. elegans.* Compared with traditional machine learning algorithms [44,45], deep learning algorithms offer many advantages. A two-way feature fusion model based on convolutional neural networks and biomarker changes was proposed to estimate the lifespan stages of *C. elegans*. The model can be divided into an EfficientNet-based sub-module with an attention mechanism and an aggregation feature extraction module that uses traditional image processing algorithms to calculate the aggregation information for fluorescent protein bright spots. The experimental results showed that, compared with other classification networks, the model introduced in this paper can effectively improve classification accuracy and meet the needs of biological research for the estimation of *C. elegans* lifespan stages.

The focus of this paper is the study of lifespan stage estimation of *C. elegans* based on convolutional neural networks and biomarker changes. The aging assessment system designed has a higher possibility of technology migration than previous studies. In the absence of interventions or restricted conditions for the cultivation of *C. elegans*, a high accuracy rate can be maintained, reflecting the aging process of *C. elegans* under natural conditions. The results for this system show that in *C. elegans* the degree of protein aggregation is a good indicator of aging, while machine learning and deep learning can maximize the use of biological data. The research results can be used as references for the future development of human lifespan evaluation and prediction methods. It should be noted that there are some deficiencies and unresolved problems with the research which need to be further explored and studied. First, for the detection of fluorescent protein bright spots of *C. elegans*, new methods could be investigated to segment the *C. elegans* images. For example, we could explore more target detection models in the computer vision field for the detection of fluorescent protein bright spots. Second, for the *C. elegans* lifespan stage estimation model based on convolutional neural networks, different levels of features could be optimized, because there is a certain amount of redundant information connecting different levels of features. Third, this paper only analyzed images containing a single *C. elegans*, and the actual application scenarios are limited. As this method relies on high-resolution images to capture fluorescent bright spots, it is currently unable to work on a large number of *C. elegans* images. In the future, we plan to investigate lifespan estimation and analysis of multiple *C. elegans* in a single image. The model framework can also be transported to mobile platforms, such as TensorFlow Lite, so that it can be applied in actual work scenarios.

## Figures and Tables

**Table 1:** Number of datasets.

Group	Number of Images	Training	Test
1	742	593	149
2	976	780	196
3	587	469	118
4	677	541	136
5	822	657	165
6	778	622	156

**Table 2:** SimpleBlobDetector parameter settings.

Parameter Type	Parameter Value
thresholdStep	8
minArea	10
maxArea	2500
minCircularity	0.2
minConvexity	0.75
minInertiaRatio	0.1

**Table 3:** Ablation experiment results for each module.

Model	AC	Recall	*F*1
*M*1	0.640	0.647	0.643
*M*2	0.526	0.542	0.534
*M*1 + *M*2	0.726	0.742	0.734

**Table 4:** Comparison of the method introduced in this paper with other classification models with respect to the *M*2 module. We performed two experiments for each model. The first row of experimental results for each model presents the results without the *M*2 module. The second row presents the experimental results using the *M*2 module.

Model	*M*2	AC	Recall	*F*1
VGG16		0.478	0.486	0.477
	✓	0.556	0.574	0.583
InceptionV3		0.554	0.574	0.569
	✓	0.602	0.614	0.615
MobileNetV3		0.511	0.529	0.513
	✓	0.571	0.593	0.580
ResNet50		0.558	0.524	0.522
	✓	0.653	0.650	0.657
DenseNet		0.575	0.557	0.565
	✓	0.713	0.713	0.723
Ours		0.640	0.647	0.643
	✓	0.726	0.742	0.734

**Table 5:** Comparison of the method introduced in this paper with other classification models with respect to the *M*2 module. The first row of experimental results presents the results without the attention module. The second row presents the experimental results using the attention module.

Model	Attention	AC	Recall	*F*1
VGG16		0.508	0.557	0.496
	✓	0.556	0.574	0.583
InceptionV3		0.543	0.596	0.548
	✓	0.602	0.614	0.615
MobileNetV3		0.583	0.595	0.566
	✓	0.571	0.593	0.580
ResNet50		0.473	0.499	0.490
	✓	0.653	0.650	0.657
DenseNet		0.666	0.702	0.698
	✓	0.713	0.713	0.723
Ours		0.609	0.703	0.714
	✓	0.726	0.742	0.734

**Table 6:** Statistical results for the random forest method.

	AC	Recall	*F*1
1	0.82	0.82	0.82
2	0.65	0.71	0.68
3	0.34	0.31	0.33
4	0.37	0.42	0.39
5	0.42	0.40	0.41
6	0.63	0.57	0.60
Overall accuracy	0.50	-	-

**Table 7:** Statistical results for the SVM method.

	AC	Recall	*F*1
1	0.54	0.26	0.35
2	0.34	0.38	0.36
3	0.17	0.04	0.07
4	0.25	0.96	0.39
5	0.00	0	0
6	0.00	0	0
Overall accuracy	0.27	-	-

## Data Availability

No new data were created or analyzed in this study. Data sharing is not applicable to this article.
